# A Bandless Richmond Crown

**Published:** 1907-06-15

**Authors:** J. C. Longfellow

**Affiliations:** Bellefontaine, Ohio


### A Bandless Richmond Crown.

BY J. C. LONGFELLOW, BELLEFONTAINE, OHIO.

There are a great many ways of constructing porcelain-faced crowns, but in the normal or average occlusion, the bandless Richmond, as we shall try to describe, will be found to be very satisfactory.

Right here we might say that by the use of porcelain in our crown work, nature has been nearer approached than by any other means.

In the construction of this crown the tooth is prepared as for a Richmond Crown. Cut a piece of pure gold about forty-gauge or heavier, what you think will cover the root with a margin all the way around, and leave a good length at lingual part. Burnish this piece over the root slightly and remove and cut hole a little smaller than for the pin to pass through, replace your gold after selecting a suitable pin, and run the pin through the gold into root as far as is necessary

Remove the gold with pin and solder with 20-k. solder, replace the pin and gold which is now in one piece, being sure to leave the pin extending out of root and gold, so when the impression is taken the pin will have something to hold to. After replacing the pin and gold, hold the pin firmly in the root, and with a flat or suitable instrument burnish the gold well on root until you can see the root margins on the gold.

Remove and trim to where root margin is marked on gold, except at lingual part, that is allowed to extend longer than the other part.

Now remove, and with the shears slit this long piece on lingual side into where you see the root margin on the gold at this part, replace and burnish or turn this back well against the lingual portion of root, letting it extend into the free margin of gum against the root. Having fitted this well against the root, we are ready to take the bite and impression. The pin and cap should readily come off with your impression. Make your cast with some investing compound and mount on articulator, select a suitable facing and grind it to the root margin, or where it shows in the gold on labial side, leaving the sides of facing extending a little beyond the margins at sides of roots.

Before grinding in your facing, the part of the pin that is extending out can be cut or ground off. After your facing has been ground in as you want it, you are ready to wax it to invest. In waxing be sure to wax out to end of gold where it extends up on the lingual portion of root. You invest and solder as usual with 18-k. solder, letting it run well upon this lingual part. Having it soldered, you can now see just where to trim and polish to, the root margin showing on the gold very plainly.

When this is mounted it is sure to fit accurately to the root it is made over, and you have practically as strong a crown as a Richmond, and no ill-fitting band extending into the gum to irritate and cause some trouble that will result in the loss of the tooth sooner or later.

Platinum can be used in the place of gold if preferred, as none of it shows.

I always use a Steele interchangeable Tooth; in case of an accident it can be easily repaired. A crown constructed in this way should last a lifetime, everything else being equal.

